# Evaluation of the surface free energy of plant surfaces: toward standardizing the procedure

**DOI:** 10.3389/fpls.2015.00510

**Published:** 2015-07-07

**Authors:** Victoria Fernández, Mohamed Khayet

**Affiliations:** ^1^Forest Genetics and Ecophysiology Research Group, Plant Physiology and Anatomy Unit, School of Forest Engineering, Technical University of MadridMadrid, Spain; ^2^Department of Applied Physics I, Faculty of Physics, Complutense University of MadridMadrid, Spain; ^3^Madrid Institute for Advanced Studies of Water (IMDEA Water Institute)Madrid, Spain

**Keywords:** contact angles, cuticle, geometric mean, plant surfaces, surface free energy, three-liquids method

## Abstract

Plant surfaces have been found to have a major chemical and physical heterogeneity and play a key protecting role against multiple stress factors. During the last decade, there is a raising interest in examining plant surface properties for the development of biomimetic materials. Contact angle measurement of different liquids is a common tool for characterizing synthetic materials, which is just beginning to be applied to plant surfaces. However, some studies performed with polymers and other materials showed that for the same surface, different surface free energy values may be obtained depending on the number and nature of the test liquids analyzed, materials' properties, and surface free energy calculation methods employed. For 3 rough and 3 rather smooth plant materials, we calculated their surface free energy using 2 or 3 test liquids and 3 different calculation methods. Regardless of the degree of surface roughness, the methods based on 2 test liquids often led to the under- or over-estimation of surface free energies as compared to the results derived from the 3-Liquids method. Given the major chemical and structural diversity of plant surfaces, it is concluded that 3 different liquids must be considered for characterizing materials of unknown physico-chemical properties, which may significantly differ in terms of polar and dispersive interactions. Since there are just few surface free energy data of plant surfaces with the aim of standardizing the calculation procedure and interpretation of the results among for instance, different species, organs, or phenological states, we suggest the use of 3 liquids and the mean surface tension values provided in this study.

## Introduction

Plant surfaces play a crucial role in protecting organs against an array of biotic and abiotic stress factors (Riederer, 2006). For instance, they may serve as barrier for the attack of pests and pathogens (Eigenbrode and Jetter, 2002; Serrano et al., 2014), high UV and visible radiation intensities (Reicosky and Hanover, 1978; Karabourniotis and Bormann, 1999), and chiefly the uncontrolled loss of water (Riederer and Schreiber, 2001). On the other hand, plant surface permeability to liquids may be a phenomenon of ecophysological and agronomic significance since it may enable the absorption of, for example, liquid water (Oliveira et al., 2005; Fernández et al., 2014a), fog (Limm and Dawson, 2010; Eller et al., 2013; Berry et al., 2014), dew (Konrad et al., 2012), foliar fertilizers (Fernández and Eichert, 2009; Fernández and Brown, 2013) or biostimulants (Saa et al., 2015). This will be first influenced by the wettability and adhesion or repellence of water drops onto plant surfaces (Fernández et al., 2014a).

Most aerial plant organs are protected by epidermal cells (including stomata and/or trichomes) of different shapes and sizes (Javelle et al., 2011; Fernández and Brown, 2013). The epidermis itself is covered with a cuticle, a layer which may be interpreted as a lipidized area of the epidermal cell wall (Guzmán et al., 2014). The outermost surface of the cuticle is the epicuticular wax layer, which may present a major degree of variability depending on the chemical and structural nature of epicuticular waxes (Barthlott et al., 1998; Jetter et al., 2006), and may also be affected by the prevailing environmental conditions during plant growth (Jeffree et al., 1975; Shepherd and Wynne Griffiths, 2006). Depending on the micro- and nano-structure of epidermal cells, epicuticular waxes and cuticular folds, plant surfaces may have different degrees of roughness and water drop repellence or adhesion (Koch and Barthlott, 2009; Fernández et al., 2014a). During the last decade and in light of the major plant surface variability observed by scanning electron microscopy (SEM) (Barthlott et al., 1998), there is a rising interest in examining plant surface topography and performance when in contact with water drops for the development of bio-mimetic materials (Bhushan and Jung, 2010). The existing epidermal micro-and nano-surface features together with surface chemical composition (Khayet and Fernández, 2012) will determine the degree of plant surface roughness and hydrophobicity (Fernández et al., 2014a).

Despite the fact that the mechanisms of foliar uptake of water and solutes by plant surfaces are still not fully understood (Fernández and Eichert, 2009; Burkhardt and Hunsche, 2013), several studies analyzed the interactions of plant surfaces with water (Brewer et al., 1991; Brewer and Smith, 1997; Hanba et al., 2004; Brewer and Nuñez, 2007; Dietz et al., 2007; Roth-Nebelsick et al., 2012; Rosado and Holder, 2013; Urrego-Pereira et al., 2013; Wang et al., 2014). Such investigations largely focussed on analysing the wettability and hydrophobicity of plant surfaces based on water contact angles. However, Fernández et al. (2011, 2014a) and Khayet and Fernández (2012) suggested the implementation of membrane science approaches for analysing the physico-chemical properties of plant surfaces and introduced surface free energy results derived from the measurement of contact angles of 3 liquids with different degrees of dispersive and non-dispersive interactions according to the method of Van Oss et al. (1987, 1988).

The surface tensions of the interfaces of a solid with liquids and the surrounding vapors are key parameters, which are commonly applied for characterizing the physico-chemical properties of surfaces in many areas of applied science and technology (Correia et al., 1997). The theory of interfacial tensions (γ) and surface free energy (*SFE*) was first introduced by Young (1805). Subsequently, several theoretical approaches were developed between the 1950's and the 1980's (e.g., Fox and Zisman, 1950; Girifalco and Good, 1957; Fowkes, 1964; Owens and Wendt, 1969; Wu, 1971; Good, 1977; Van Oss et al., 1986, 1988) with the aim of estimating solid surface tension (γ_*s*_) or *SFE* which is the same) from contact angles of 2 or 3 liquids with different dispersive and non-dispersive components.

Despite the interest in estimating the *SFE* of different solids ranging from polymers and co-polymer blends (e.g., Correia et al., 1997; Adão et al., 1999), to polymeric membranes (Khayet et al., 2003, 2007), geological materials (Chibowski and Staszczuk, 1988; Helmy et al., 2004), or wood (Gindl et al., 2001), there is a fair degree of controversy concerning several associated aspects such as the liquid surface tension and contact angle determination methods, the use of 2, 3 or more test liquids and the different calculation methods reported (Morra, 1996; Della Volpe and Siboni, 1997; Jañczuk et al., 1999; Kwok and Neumann, 1999; Tretinnikov, 2000; Chibowski and Perea-Carpio, 2002; Della Volpe et al., 2004).

Since there are still few *SFE* data of plant materials (Gorb et al., 2004; Fernández et al., 2011, 2014a,b; Khayet and Fernández, 2012; Wang et al., 2014), and current contact angle measuring devices are available with software enabling: (i) the direct estimation of the *SFE* of solids and their components according to different methods, and (ii) the selection of different test liquid surface tension values, we carried out preliminary efforts to revise the existing *SFE* theory together with the most commonly used *SFE* determination methods, and to calculate the mean values for the total surface tension and surface tension components of water (W), glycerol (G) and diiodomethane (DM), which have been often used for such purpose. It must be stressed that *SFE* estimations of a solid surface by contact angle measurements of either 2 or 3 test liquids of the same nature should be similar or at least within the same range as reported in several studies performed with some synthetic materials (e.g., Adão et al., 1999; Khayet et al., 2003). However, the major structural (roughness) and chemical (polarity, apolarity and hydrogen-bonding interactions) variation observed among plant species, organs or organ parts made us develop trials to calculate the *SFE* of model plant surfaces when measured with the most commonly used *SFE* estimation methods, which employ either 2 or 3 test liquids. With the aim of choosing a procedure for determining the *SFE* of plant materials to be systematically used in future research attempts, we further calculated this parameter using contact angle measurements of either pairs of liquids (i.e., W-DM, G-DM, W-G) considering both the geometric and harmonic mean approaches, or 3 liquids (W-G-DM) applying the Lifshitz-van der Waals-acid-base or van Oss, Good and Chaudhury method (Van Oss et al., 1986, 1987, 1988). The contact angles of W, G and DM were determined on to 3 rough and 3 rather flat model plant surfaces and the *SFE* obtained by different methods were compared.

## Materials and methods

### Plant material

The plant materials analyzed as model correspond to intact, mature red ironbark (*Eucalyptus sideroxylon* A. Cunn. ex Woolls) leaves, juvenile blue gum eucalypt (*Eucalyptus globulus* Labill.) leaves, mature Chilean myrtle (*Luma apiculata* (DC.) Burret) leaves, mature rubber tree (*Ficus elastica* Roxb. ex Hornem) leaves, mature holm-oak (*Quercus ilex* subsp. *ballota* (Desf.) Samp.) leaves, and red bell peppers (*Capsicum annum* L. cv. “Genil”). Rubber tree and Chilean myrtle leaves and also pepper fruit surfaces are smooth while red iron bark, blue gum eucalypt and holm oak leaf surfaces are extremely rough. We examined the properties of the upper leaf side of red ironbark blue gum eucalypt, Chilean myrtle, and rubber tree. The lower leaf side of holm-oak and the pepper fruit surface were also analyzed.

### Scanning electron microscopy

Gold sputtered plant materials were examined with a Hitachi S-3400 N (Tokyo, Japan) and a Philips XL30 (Eindhoven, The Netherlands) scanning electron microscope (SEM).

### Contact angle measurements

The test liquids analyzed were double-distilled water (W), glycerol (G) and diiodomethane (DM, both 99% purity, Sigma-Aldrich). Advancing contact angles of drops of such liquids were measured at 20°C using a contact angle meter CAM 200 (KSV Instruments Ltd., Helsinki, Finland). Drops of each liquid were deposited on to intact plant surfaces (30 repetitions) with a manual dosing system holding a 1 mL syringe (0.5 mm diameter needle). Side view images of the drops were captured at a rate of 10 frames s^−1^. Contact angles were automatically calculated by fitting the captured drop shape to the one calculated from the Young–Laplace equation.

### Surface free energy calculation methods

Since the total *SFE*, γ_*s*_, cannot be calculated directly (Khayet et al., 2003), different indirect methods have been proposed which based on the contact angle measurement of either 2 or 3 different liquids deposited on to a solid surface (Jañczuk and Białopiotrowicz, 1989; Della Volpe and Siboni, 1997). Three calculation methods were used for estimating γ_*s*_, its components and the resulting solubility parameter (δ) of different plant materials. Provided that we observed a significant dispersion of the total surface tension and surface tension component data provided in the literature for various liquids including W, G and DM, the mean values of all results gathered were considered in this study (Table 1).

**Table 1:** **Total surface free energy or surface tension (γ_*l*_) and its components (measured at 20°C) of water (W), Glycerol (G), and diiodomethane (DM)**.

		***Liquid* γ components) for GM and HM methods (mJ m^−2^)**		***Liquid* γ components for 3-L method (mJ m^−2^)**		
**Liquid**	**γ_*l*_ (mJ m^−2^)**	**γ^*d*^**	**γ^*nd*^**	**γ^*LW*^**	**γ^+^**	**γ^−^**
W	72.8 ± 0.0^a^	21.8 ± 0.7^a^	51.0 ± 0.7^a^	21.8^c^	25.50^c^	25.50^c^
G	63.7 ± 0.4^b^	33.6 ± 0.3^b^	30.1 ± 0.4^b^	33.6 ± 0.3^d^	8.41 ± 3.02^d^	31.16 ± 14.23^d^
DM	50.8 ± 0.0^a^	49.0 ± 0.5^a^	1.8 ± 0.5^a^	50.8 ± 0.0^d^	0.56 ± 0.50^d^	0.00 ± 0.00^d^

*For calculations following the GM and HM methods (dispersive (γ^d^) and non-dispersive (γ^nd^) components) and for the 3-L method (Lifshitz-van der Waals (γ^LW^) and acid-base (γ^AB^; γ^+^ and γ^−^) components). Data are means ± SD*.

a *Mean values calculated from Fowkes (1964), Owens and Wendt (1969), Wu (1971, 1982) and Jañczuk and Białlopiotrowicz (1989)*.

b *Mean values calculated from Fowkes (1964), Dann (1970), Panzer (1973), Wu (1971, 1982), Jañczuk and Chibowski (1983) and Jañczuk et al. (1993)*.

c *Reference values taken by Van Oss et al. (1987)*.

d *Mean values calculated from van Oss (1994) and Jañczuk et al. (1993, 1999)*.

### Calculations based on 2 liquids

The 2-liquid based, geometric mean and harmonic mean methods of calculation of *SFE* are commonly used for characterizing synthetic surfaces (e.g., Correia et al., 1997; Adão et al., 1999) and have been used in this investigation.

Based on the theory of Girifalco and Good (1957), Fowkes (1962, 1964) proposed that γ_*s*_ (*SFE* of the solid to be analyzed) is the sum of contributions from the different intramolecular forces at the surface: those due to dispersion interactions, dipole-dipole interactions, dipole induced-dipole interactions, hydrogen bonding, Π-bonding, electrostatic interactions and acceptor-donor interactions.

For simplicity, γ_*s*_ ca be expressed as the sum of dispersive (γ^*d*^_*s*_) and non-dispersive (γ^*nd*^_*s*_) interactions (Jañczuk et al., 1989; Kwok et al., 1994):

(1) $$\gamma_{s}=\gamma_{s}^{d}+\gamma_{s}^{nd}$$

While there is agreement in the nomenclature for the dispersive *SFE* component (γ^*d*^_*s*_), many authors referred to the remaining term (i.e., γ^*nd*^_*s*_), which obviously includes many types of forces (Fowkes, 1964), as polar component γ^*p*^_*s*_ (e.g., Wu, 1971). To avoid any possible misinterpretation, we will subsequently refer to the γ^*d*^_*s*_ and γ^*nd*^_*s*_ components throughout the manuscript.

### Geometric mean method

Based on Fowkes' assumption that dispersive interactions between the molecules of the solid and the liquid prevailed among other acting forces, and considering Young's equation, the following expression was suggested (Fowkes, 1964):

(2) $$2\sqrt{\gamma_{s}^{d}\gamma_{l}^{d}}=\gamma_{l}(1+\cos\theta )$$

where, γ_*l*_ is the total surface tension of the liquid, γ^*d*^_*l*_ is the dispersive component of the liquid and θ is the contact angle measured between the liquid and the solid under study.

This equation was further expanded by Owens and Wendt (1969) to:

(3) $$2\sqrt{\gamma_{s}^{d}\gamma_{l}^{d}}+2\sqrt{\gamma_{s}^{nd}\gamma_{l}^{nd}}=\gamma_{l}(1+\cos\theta )$$

Where, γ^*nd*^_*l*_ is the non-dispersive component of the liquid and γ^*nd*^_*s*_ is the non-dispersive component of the solid.

Equation (3) is known as the Geometric mean, *SFE* calculation method, termed hereafter GM method.

### Harmonic mean method

Wu (1971) proposed an alternative equation, based on the reciprocal mean and force additivity, which once applied to Young's equation for two liquids results in:

(4) $$4\left(\frac{\gamma_{s}^{d}\gamma_{l}^{d}}{\gamma_{s}^{d}+\gamma_{l}^{d}}+\frac{\gamma_{s}^{nd}\gamma_{l}^{nd}}{\gamma_{s}^{nd}+\gamma_{l}^{nd}}\right)=\gamma_{l}(1+\cos\theta )$$

Equation (4) is known as the Harmonic mean, *SFE* calculation method, termed hereafter HM method.

### Three-liquids method

According to the Lifshitz-van der Waals-acid-base method, or van Oss, Good, and Chaudhury method (Van Oss et al., 1986, 1987, 1988) γ can be divided into the following components:

(5) $$\gamma_{i}=\gamma_{i}^{LW}+\gamma_{i}^{AB}=\gamma_{i}^{LW}+2\sqrt{\gamma_{i}^{+}\gamma_{i}^{-}}$$

where *i* denotes either the solid or the liquid phase and the acid-base component (γ^*AB*^) breaks down into the electron-donor (γ^−^) and the electron-acceptor (γ^+^) interactions. For a solid-liquid system, the following expression is given (Van Oss et al., 1987, 1988):

(6) $$2{\left(\gamma_{s}^{LW}\gamma_{l}^{LW}\right)}^{1/2}+2{\left(\gamma_{s}^{+}\gamma_{l}^{-}\right)}^{1/2}+2{\left(\gamma_{s}^{-}\gamma_{l}^{+}\right)}^{1/2}=\gamma_{l}(1+\cos\theta )$$

where the 3 surface tension components (i.e., γ^*LW*^_*s*_, γ^+^_*s*_ and γ^−^_*s*_) together with the *SFE* of the solid measured (γ_*s*_) can be obtained. This *SFE* calculation procedure is termed hereafter 3-L method.

### Solubility parameter calculations

From the total surface tension and surface tension components estimated by the methods described above, the solubility parameter (δ) of plant surfaces was calculated using the following relation (Khayet et al., 2002):

(7) δ=(ec)12

where *e*_*c*_ (MJ m^−3^) is the cohesive energy density, which is related to γ_*s*_ (mJ m^−2^) as follows:

(8) ec=(γs0.75)32

## Results

### Liquid surface tension mean values

The average values of the total surface tension γ_*l*_ and its components for the 3 liquids measured (W, G, and DM) were calculated together with their standard deviation using the data reported in the literature. For the GM and the HM, 2-liquids methods, the surface tension components are γ^*d*^_*l*_ and γ^*nd*^_*l*_ while for the 3-L method they are γ^*LW*^, γ^−^, and γ^+^ (Table 1). The estimation of γ^*d*^_*l*_ either via measuring contact angles on to a known solid or by liquid-liquid interfacial tension measurements may differ (Jañczuk et al., 1993). This was chiefly noticeable in the case of G.

Beginning with the 2-liquid approaches (i.e., the GM and HM methods), the mean γ_*l*_, γ^*d*^_*l*_, and γ^*nd*^_*l*_ values of W and DM were obtained from: Fowkes (1964), Owens and Wendt (1969), Wu (1971, 1982), and Jañczuk and Białlopiotrowicz (1989). Glycerol surface values are the average of the results reported by Fowkes (1964), Dann (1970), Panzer (1973), Wu (1971, 1982), Jañczuk and Chibowski (1983) and Jañczuk et al. (1993).

The Liftshizt-van der Waals and acid-base components (including γ^−^ and γ^+^) of W were taken as reference by Van Oss et al. (1987). The γ^*LW*^, γ^−^ and γ^+^ mean values of G and DM were calculated from the data reported by van Oss (1994) and Jañczuk et al. (1993, 1999). For G, it is remarkable the high degree of dispersion of the γ^+^ and chiefly the γ^−^ components (see Table 1).

### Plant surface topography

**Figure 1:**
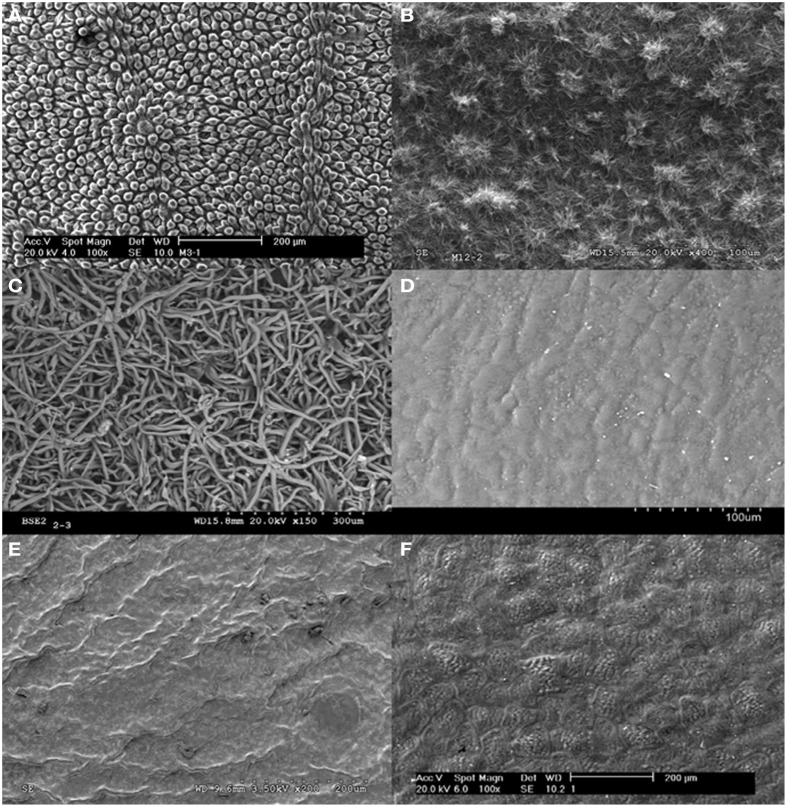
**Plant surfaces analysed. (A)** Upper leaf side of red ironbark (major micro- and nano roughness), **(B)** upper leaf side of juvenile blue gum (major micro- and nano roughness conferred by wax nano-tubes), **(C)** lower leaf side of holm oak (great micro-roughness conferred by the hairs/trichomes), **(D)** upper leaf side of rubber tree (rather smooth), **(E)** upper leaf side of Chilean myrtle (rather smooth), and **(F)** pepper fruit surface (rather smooth).

The adaxial leaf surface of red ironbark and blue gum eucalypt, and abaxial leaf side of holm oak were found to have a high degree of roughness (Figures 1A–C). This is due to the presence of milimetric trichomes on to the lower leaf side of holm-oak, wax nano-tubes on the surface of blue gum eucalypt leaves, and wax platelets and a complex micro-topography provided by the epidermal cells of red ironbark leaves. By contrast, the upper leaf surface of rubber tree and Chilean myrtle is rather flat alike that of pepper fruit (Figures 1D–F).

### Contact angles

**Figure 2:**
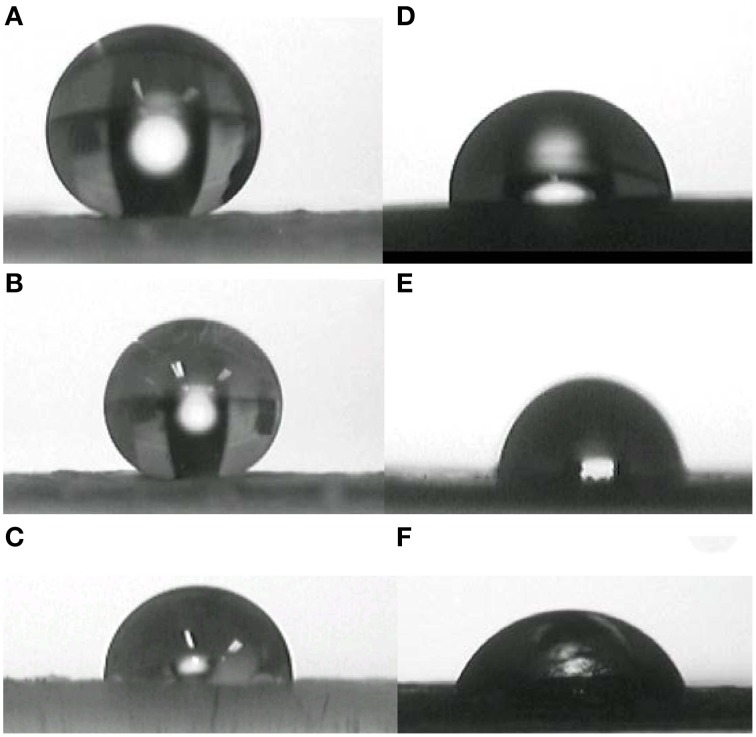
**Contact angles of water (A,D), glycerol (B,E) and diiomethane (C,F) on to blue gum (rough surface; A–C) and pepper fruit (smooth surface; D–F) surfaces, as an example**.

The contact angle values of W, G, and DM with the surface of the examined plant materials are shown in Table 2. In Figure 2 the contact angles of water (W), glycerol (G), and diiodomethane (DM) with one of the rough (blue gum leaf) and smooth (pepper fruit) surfaces analyzed are shown as an example. The structural complexity observed in the adaxial surfaces of red ironbark, blue gum eucalypt, and abaxial holm-oak leaf surfaces led to very high contact angles with polar liquids (i.e., W and G; Figures 2A,B). While the blue gum eucalypt leaf is wettable (θ < 90°) for DM (Figure 2C), which suggests the occurrence of chemical interactions between such liquid and epicuticular wax nano-tubes, the surfaces of red ironbark and holm oak leaves are unwettable (θ > 90°) for such largely apolar liquid.

**Table 2:** **Contact angles of water (θ**_***w***_**), glycerol (θ**_***g***_**) and diiodomethane (θ**_***d***_**) on the upper side of red ironbark, blue gum eucalypt, rubber tree and Chilean myrtle leaves, lower side of holm oak leaves, and pepper fruit surfaces**.

**Sample**	**θ_*w*_ (°)**	**θ_*g*_ (°)**	**θ_*d*_ (°)**
Red ironbark	138.29 ± 4.39	144.68 ± 7.25	126.07 ± 3.87
Blue gum eucalypt	142.58 ± 6.70	136.52 ± 11.15	84.03 ± 6.99
Holm oak	134.77 ± 4.85	139.08 ± 4.55	123.09 ± 3.04
Rubber tree	83.75 ± 8.19	82.03 ± 7.51	59.17 ± 3.94
Chilean myrtle	100.48 ± 4.97	98.24 ± 6.49	60.01 ± 2.65
Pepper fruit	83.39 ± 4.72	68.57 ± 9.23	60.80 ± 6.24

Pepper fruit and rubber gum leaf surfaces are wetted by W, G and DM drops (see Figures 2D–F as an example). The surface of Chilean myrtle is however unwettable for W and G, but the contact angles measured (approximately 100°) are well below those determined for red ironbark, blue gum eucalypt, and holm oak leaves.

### Surface free energy calculation by different procedures

The *SFE* results calculated for the mean surface tension values (Table 1) and contact angles (Table 2) according to the 3-L, GM or HM methods are grouped in Tables 3–**8** for the different plant materials assessed. It must be highlighted that the 3 model smooth surfaces analyzed had lower contact angles chiefly with polar liquids and higher *SFE* values, which were within a similar range (approximately from 31 to 38 mJ m^−2^) at least when determined by the 3-L method. The rough surfaces examined had very high contact angles with polar liquids (see Figure 2 as an example) and *SFE* varying from 5 to 19 mJ m^−2^ when calculated by the 3-Liquids method.

**Table 3:** **Surface free energy (γ**_***s***_**), solubility parameter (δ), surface free energycomponents, i.e., Lifschitz-van der Waals or dispersive component (γ^LW or d^**_***s***_**) acid-base or non-dispersive component (γ^AB or nd^**_***s***_**), electron-donor (γ^−^**_***s***_**) and the electron-acceptor (γ^+^**_***s***_**) components), of adaxial red ironbark leaf surfaces calculated by the 3-liquids (3-L), geometric mean (GM) and harmonic mean (HM) methods**.

**Method**	**Test liquids**	**γ^LW or d^_*s*_ (mJ m^−2^)**	**γ^−^_*s*_ (mJ m^−2^)**	**γ^+^_*s*_ (mJ m^−2^)**	**γ^AB or nd^_*s*_ (mJ m^−2^)**	**γ_*s*_ (mJ m^−2^)**	**δ(MJ^1/2^ m^−3/2^)**
3-L	W, G, DM	1.60	3.67	1.58	4.82	6.42	5.01
GM	W, DM	2.02 (26.3%)	–	–	0.13 (−97.3%)	2.16 (−66.4%)	2.21 (−55.9%)
GM	G, DM	2.61 (63.1%)	–	–	0.41 (−91.5%)	3.01 (−53.1%)	2.84 (−43.3%)
GM	W, G	0.31 (−80.6%)	–	–	0.86 (−82.2%)	1.17 (−81.8%)	1.40 (−72.1%)
HM	W, DM	5.83 (264.4%)	–	–	0.02 (−99.6%)	5.84 (−9.0%)	4.66 (−7.0%)
HM	G, DM	*	–	–	*	*	*
HM	W, G	*	–	–	*	*	*

*The deviation from the 3-L method is indicated in brackets*.

*Values cannot be calculated*.

Since we understand that the characterization of surfaces by measuring contact angles of 3 liquids with different dispersive and non-dispersive contributions (W, G, and DM) provides more information than comparing pairs of liquids (W-DM, G-DM, or W-G), we analyzed the *SFE* results taking the 3-L method as reference. Furthermore, since we used average surface tension and surface tension component values, which are comparable for all the *SFE* estimation approaches employed, it could be expected that, for the same plant material, at least some of the *SFE* results derived from pairs of liquids would be within the range obtained by the 3-L method. Based on the developed theory (Equations 3–6) it would be desirable that data estimated for γ^LW^_*s*_ and γ^*d*^_*s*_, γ^AB^_*s*_ and γ^nd^_*s*_, and also γ_*s*_ and δ would be comparable among methods. For assessing the potential similarity between the results derived from 2 and 3 liquid calculation procedures, the percentage deviation from de 3-L method is provided in Tables 3–**8**.

The upper leaf surface of red iron bark, which is quite rough (Figure 1A), was found to have quite low total *SFE* (6.42 mJ m^−2^) and δ (5.01 MJ^½^ m^−3/2^) values which are however within the range determined for holm oak abaxial surfaces. The γ^LW^_*s*_ is lower than the γ^AB^_*s*_ component chiefly due to the high γ^−^_*s*_ value (Table 3). While selecting W-DM data and calculating the *SFE* by the HM method, γ_*s*_ and δ values do not deviate so much for the 3-L method results (−9.0 and 7.0% deviation, respectively). However, this approach led to a major over-estimation of the γ^LW^_*s*_ component. The remaining GM and HM results have a high degree of dispersion when compared to the 3-L method.

The adaxial side of juvenile blue gum leaves has also a high degree of roughness (Figure 1B). The total *SFE* calculated by the 3-L method is 19.07 mJ m^−2^, principally due to a large γ^LW^_*s*_ compared to the γ^AB^_*s*_ component (Table 4). The *SFE* and δ results obtained with W-DM and the GM method had the lowest deviation (from 12 to 27% for all parameters) from the 3-L method. The remaining results obtained with the GM and HM methods led to significant *SFE* and δ under or over-estimations in relation to the 3-L method values.

**Table 4:** **Surface free energy (γ**_***s***_**) and its components (γ^LW or d^**_***s***_ **γ^AB or nd^**_***s***_**, γ^+^**_***s***_ **and γ^−^**_***s***_**), and solubility parameter (δ) of adaxial blue gum eucalypt leaf surfaces calculated by the 3-L, GM, and HM methods**.

**Method**	**Test liquids**	**γ^LW or d^_*s*_ (mJ m^−2^)**	**γ^−^_*s*_ (mJ m^−2^)**	**γ^+^_*s*_ (mJ m^−2^)**	**γ^AB or nd^_*s*_ (mJ m^−2^)**	**γ_*s*_ (mJ m^−2^)**	**δ (MJ^1/2^ m^−3/2^)**
3-L	W, G, DM	14.87	0.55	7.98	4.22	19.07	11.32
GM	W, DM	18.92 (27.2%)	–	–	3.22 (−23.7%)	22.14 (16.1%)	12.67 (11.9%)
GM	G, DM	21.53 (44.8%)	–	–	10.95 (159.5%)	32.49 (70.4%)	16.88 (49.1%)
GM	W, G	1.82 (−87.8%)	–	–	0.03 (−99.3%)	1.85 (−90.3%)	1.97 (−82.6%)
HM	W, DM	9.02 (−39.3%)	–	–	−2.50 (−159.2%)	6.51 (−65.9%)	5.06 (−55.3%)
HM	G, DM	9.00 (−39.5%)	–	–	−2.50 (−159.2%)	6.50 (−65.9%)	5.05 (−55.4%)
HM	W, G	8.91 (−40.1%)	–	–	−2.46 (−158.3%)	6.46 (−66.1%)	5.03 (−55.6%)

*The deviation from the 3-L method is indicated in brackets*.

The lower leaf surface of holm oak has a high degree of micro-roughness provided by pubescence (Figure 1C), and low total *SFE* (5. 97 mJ m^−2^) and δ (4.74 MJ^½^ m^−3/2^) values (Table 5). When considering W-DM data and using the HM method, γ_*s*_ and δ values presented a low deviation from the 3-L method (11.1 and 8.2%, respectively). However, the HM approach led to a major over-estimation of γ^d^_*s*_ (189.1%) and an under-estimation (−80.0%) of the γ^*nd*^_*s*_ components, as also recorded for red ironbark (Table 3). The remaining GM and HM values significantly deviated from the results obtained using the 3-L method (Table 5).

**Table 5:** **Surface free energy (γ**_***s***_**) and its components (γ^LW or d^**_***s***_ **γ^AB or nd^**_***s***_**, γ^+^**_***s***_ **and γ^−^**_***s***_**), and solubility parameter (δ) of abaxial holm oak leaf surfaces calculated by the 3-L, GM and HM methods**.

**Method**	**Test liquids**	**γ^LW or d^_*s*_ (mJ m^−2^)**	**γ^−^_*s*_(mJ m^−2^)**	**γ^+^_*s*_ (mJ m^−2^)**	**γ^AB or nd^_*s*_ (mJ m^−2^)**	**γ_*s*_ (mJ m^−2^)**	**δ (MJ^1/2^ m^−3/2^)**
3-L	W, G, DM	2.02	3.50	1.11	3.95	5.97	4.74
GM	W, DM	2.41 (19.3%)	–	–	0.24 (−93.9%)	2.65 (−55.6%)	2.58 (−45.6%)
GM	G, DM	2.98 (47.5%)	–	–	0.16 (−95.9%)	3.14 (−47.4%)	2.93 (−38.2%)
GM	W, G	0.05 (−97.5%)	–	–	1.86 (−52.9%)	1.91 (−68.0%)	2.01 (−57.6%)
HM	W, DM	5.84 (189.1%)	–	–	0.79 (−80.0%)	6.63 (11.1%)	5.13 (8.2%)
HM	G, DM	*	–	–	*	*	*
HM	W, G	−8.72 (−531.7%)	–	–	32.65 (726.6%)	23.93 (300.8%)	13.43 (183.3%)

*The deviation from the 3-L method is indicated in brackets*.

*Values cannot be calculated*.

The upper leaf surface of rubber tree is rather smooth (Figure 1D) and has a higher total *SFE* (principally associated with a high γ^LW^_*s*_ and a significant γ^−^_*s*_component) and δ value (31.76 mJ m^−2^ and 16.60 MJ^½^ m^−3/2^, respectively). The lowest deviation from the results obtained by the 3-L method was obtained for W-DM and the GM approach (from 2.0 to 3.9% deviation for γ^*d*^_*s*_, γ_*s*_, and δ), which however led to a 24.3% under-estimation of γ^nd^_*s*_ (Table 6). Using W-DM and G-DM pairs and calculating γ_*s*_ by the GM or HM approaches led to results with low deviations from the 3-L method, with the exception of the γ^nd^_*s*_ component which was under-estimated in the case of W-DM and largely G-DM using the GM method (−24.3 and −85.9%, respectively), and G-DM calculated with the HM method (−60.5% deviation).

**Table 6:** **Surface free energy (γ**_***s***_**) and its components (γ^LW or d^**_***s***_ **γ^AB or nd^**_***s***_**, γ^+^**_***s***_ **and γ^−^**_***s***_**), and solubility parameter (δ) of adaxial rubber tree leaf surfaces calculated by the 3-L, GM and HM methods**.

**Method**	**Test liquids**	**γ^LW or d^_*s*_ (mJ m^−2^)**	**γ^−^_*s*_(mJ m^−2^)**	**γ^+^_*s*_ (mJ m^−2^)**	**γ^AB or nd^_*s*_ (mJ m^−2^)**	**γ_*s*_ (mJ m^−2^)**	**δ (MJ^1/2^ m^−3/2^)**
3-L	W, G, DM	24.41	18.35	0.74	7.36	31.76	16.60
GM	W, DM	25.36 (3.9%)	–	–	5.57 (−24.3%)	30.93 (−2.6%)	16.27 (−2.0%)
GM	G, DM	28.02 (14.8%)	–	–	1.04 (−85.9%)	29.05 (−8.5%)	15.53 (−6.4%)
GM	W, G	5.66 (−76.8%)	–	–	16.78 (128.0%)	22.44 (−29.3%)	12.79 (−23.0%)
HM	W, DM	27.70 (13.5%)	–	–	9.46 (28.5%)	37.16 (17.0%)	18.68 (12.5%)
HM	G, DM	28.69 (17.5%)	–	–	2.91 (−60.5%)	31.61 (−0.5%)	16.54 (−0.4%)
HM	W, G	6.88 (−71.8%)	–	–	21.16 (187.5%)	28.03 (−11.7%)	15.12 (−8.9%)

*The deviation from the 3-L method is indicated in brackets*.

The rather flat upper leaf surface of Chilean myrtle (Figure 1E) has the highest total *SFE* (chiefly due to a high γ^LW^_*s*_ and a significant γ^−^_*s*_ component) and δ values of all the plant materials analyzed (38.12 mJ m^−2^ and 19.04 MJ^½^ m^−3/2^, respectively). The lowest γ^*d*^_*s*_, γ_*s*_, and δ deviations from the 3-L method were obtained for G-DM using the GM method, followed by W-DM calculated by the HM and GM approaches (Table 7). However, all of these results based on pairs of liquids led to a major under-estimation of the γ^nd^_*s*_ component (ranging from −80 to −96% deviations).

**Table 7:** **Surface free energy (γ**_***s***_**) and its components (γ^LW or d^**_***s***_ **γ^AB or nd^**_***s***_**, γ^+^**_***s***_ **and γ^−^**_***s***_**), and solubility parameter (δ) of adaxial Chilean myrtle leaf surfaces calculated by the 3-L, GM, and HM methods**.

**Method**	**Test liquids**	**γ^LW or d^_*s*_ (mJ m^−2^)**	**γ^−^_*s*_(mJ m^−2^)**	**γ^+^_*s*_ (mJ m^−2^)**	**γ^AB or nd^_*s*_ (mJ m^−2^)**	**γ_*s*_ (mJ m^−2^)**	**δ (MJ^1/2^ m^−3/2^)**
3-L	W, G, DM	25.02	10.70	4.01	13.10	38.12	19.04
GM	W, DM	28.19 (12.7%)	–	–	0.49 (−96.3%)	28.67 (−24.8%)	15.38 (−19.2%)
GM	G, DM	31.68 (26.6%)	–	–	0.94 (−92.8%)	32.62 (−14.4%)	16.94 (−11.0%)
GM	W, G	4.04 (−83.9%)	–	–	8.15 (−37.5%)	12.19 (−68.0%)	8.09 (−57.5%)
HM	W, DM	28.38 (13.4%)	–	–	2.69 (−79.5%)	31.07 (−18.5%)	16.33 (−14.2%)
HM	G, DM	^*^	–	–	^*^	^*^	^*^
HM	W, G	4.51 (−82.0%)	–	–	14.27 (8.9%)	18.77 (−50.8%)	11.19 (−41.2%)

*The deviation from the 3-L method is indicated in brackets*.

* *Values cannot be calculated*.

Finally, the rather smooth pepper fruit surface (Figure 1F) has also high γ_*s*_ (principally associated with a large γ^LW^_*s*_ component) and δ values (31.66 mJ m^−2^ and 16.56 MJ^½^ m^−3/2^, respectively). Using W-DM contact angles following the GM approach led to γ_*s*_ and δ data with low deviations from the 3-L method (with 3.1–7.4% under-estimations for γ^*d*^_*s*_, γ_*s*_ and δ and 11.4% over-estimation for γ^nd^_*s*_; Table 8). For W-G, and the GM method, γ_*s*_ and δ results slightly deviated from the 3-L method (8–18% over-estimations for γ^*d*^_*s*_, γ_*s*_ and δ and 23.2% under-estimation of γ^nd^_*s*_). Using G-DM and W-DM with the GM method, and even W-DM, G-DM and W-G with the HM approach also led to γ^*d*^_*s*_, γ_*s*_ and δ values with low deviations. However, such 2-liquid combinations led to a major over-estimation of the γ^nd^_*s*_ component (from 40 to 100%) as compared to the results obtained by the 3-L method.

**Table 8:** **Surface free energy (γ**_***s***_**) and its components (γ^LW or d^**_***s***_ **γ^AB or nd^**_***s***_**, γ^+^**_***s***_ **and γ^−^**_***s***_**), and solubility parameter (δ) of pepper fruit surfaces calculated by the 3-L, GM, and HM methods**.

**Method**	**Test liquids**	**γ^LW or d^_*s*_ (mJ m^−2^)**	**γ^−^_*s*_(mJ m^−2^)**	**γ^+^_*s*_ (mJ m^−2^)**	**γ^AB or nd^_*s*_ (mJ m^−2^**	**γ_*s*_ (mJ m^−2^)**	**δ (MJ^1/2^ m^−3/2^)**
3-L	W, G, DM	26.22	3.00	2.47	5.44	31.66	16.56
GM	W, DM	24.28 (−7.4%)	–	–	6.06 (11.4%)	30.33 (−4.2%)	16.04 (−3.1%)
GM	G, DM	23.67 (−9.7%)	–	–	7.75 (42.5%)	31.42 (−0.8%)	16.47 (−0.5%)
GM	W, G	30.95 (18.0%)	–	–	4.18 (−23.2%)	35.14 (11.0%)	17.91 (8.2%)
HM	W, DM	26.92 (2.7%)	–	–	9.84 (80.9%)	36.76 (16.1%)	18.52 (11.8%)
HM	G, DM	26.99 (2.9%)	–	–	8.73 (60.5%)	35.72 (12.8%)	18.13 (9.5%)
HM	W, G	22.91 (−12.6%)	–	–	11.11 (104.2%)	34.02 (7.5%)	17.48 (5.6%)

*The deviation from the 3-L method is indicated in brackets*.

Calculations by the HM method led to some negative results for blue gum and holm oak leaves or to no results in the case of G-DM and G-W combinations for red iron bark and also for G-DM calculations for Chilean myrtle and holm oak. These results have no physical significance and are related to mathematical constraints associated with HM method equations.

## Discussion

The *SFE* of a material is a fundamental property that determines its surface and interfacial performance in processes like wetting and adhesion (Adão et al., 1999). Despite its importance for characterizing contact phenomena related to plant surfaces, such as the interaction of plant organs with surface deposited water (Brewer et al., 1991) or insects (Prüm et al., 2012), only few studies considered this parameter in a plant science context so far (Gorb et al., 2004; Fernández et al., 2011, 2014a,b; Khayet and Fernández, 2012; Wang et al., 2014). The estimation of the *SFE* and solubility parameter of plant materials may be, for example, useful for predicting water-plant surface interactions and the potential of plant organs to absorb water and agrochemical solutions (Fernández et al., 2011, 2014a,b).

Several *SFE* calculation methods were introduced during the last 50 years and the drawbacks of estimating *SFE* based on contact angle measurements by different procedures have been highlighted in some reports (e.g., Kwok and Neumann, 1999; Chibowski and Perea-Carpio, 2002; Della Volpe et al., 2004), which may be minimized if carefully considering the procedures, at least for comparing between different plant materials, which is the aim of this study.

Surface roughness can affect liquid contact angles and it is a matter of interest for many researchers dealing with solid-liquid interactions (Bhushan and Nosonovsky, 2010). According to the models of Wenzel (1936) and Cassie and Baxter (1944), there are two regimes of wetting of a rough surface: a homogeneous regime with a 2-phase solid–liquid interface, and a non-homogeneous regime with a 3-phase solid–water–air interface (with air pockets between the solid and the liquid).

When revising the existing *SFE*-related multi-disciplinary literature, we recognized that an array of test liquids with different total surface tension and surface tension components were available. We focused on water, glycerol and diiodomethane which are commonly used due to their distinct dispersive and non-dispersive components (e.g., Adão et al., 1999; Jañczuk et al., 1999). It is worth highlighting that for test liquids, we recognized that different surface tension values were used by authors when calculating *SFE* for instance, by the GM vs. the HM approach and that the method of determination of the dispersive component (e.g., with contact angles or interfacial tension measurements) may also have an influence on the surface tension values of liquids. Additionally, we actually noticed that the calculation methods were extremely sensitive to slight surface tension and surface tension component value modifications. Therefore, we decided to work with average surface tension and surface tension component values, which may be comparable among different *SFE* calculation methods as shown in Table 1. This choice enabled us to trust that for the same plant surface, the potential *SFE* differences are not due to the different surface tension values of test liquids, but rather to the particular calculation method in combination with the surface features of the materials analyzed. Thereby, we standardized the surface tension values of W, G and DM by using the mean values calculated after revising the exiting literature, and subsequently used them for estimating the *SFE* of plant materials.

Aware that any of the SFE calculation methods based on either 2 or 3 liquids analyzed produced certain *SFE* and *SFE* component values, we wondered if the results were meaningful and comparable between and within species. For verifying this aspect, 3 rough and 3 smother plant materials were selected as model species, and contact angles with W, G, and DM were carefully measured under similar conditions.

Since we understand that the characterization of a surface by measuring drops of 3 test liquids with different properties is more informative than using only 2 liquids having different dispersive and non-dispersive interactions, we took the results obtained using the 3-L method as reference and estimated the potential deviation of *SFE*, *SFE* components and solubility parameter (δ) values. This last parameter was theoretically calculated by Khayet and Fernández (2012) for model wax compounds, and provides insight into the combined effect of surface chemical composition (plant surfaces are generally covered with waxes having a theoretical δ ranging between 16 and 17 MJ^½^ m^−3/2^) and roughness. Looking at the mean surface tension components shown in Table 1, it can be derived that in a decreasing order, W has the highest non-dispersive component, followed by G and DM. On the contrary, DM has the highest dispersive (apolar) component, followed by G and W. It is hence easy to understand that drops of such liquids may perform differently depending on the chemical and structural nature of every different surface, as observed in this study. While measuring contact angles of drops of 2 liquids may save time when determining *SFE* of samples either by the GM or HM methods, we must be sure that results are meaningful and comparable with those obtained by the 3-L method.

We could not find a clear relationship between surface roughness and *SFE* calculation methods, since for example, for the blue gum eucalypt leaf which is quite rough, W-DM contact angles estimated by the GM method provided fair *SFE* and δ estimations. On the contrary, for the rather smooth Chilean myrtle leaf, none of the pairs of liquids combinations and 2-liquid calculation methods lead to values within the range obtained with the 3-L method, chiefly due to a severe under-estimation of γ^nd^_*s*_. Our observations agree with the comments of Kwok and Neumann (1999), who noted that “there are as yet no general criteria to answer the question of how smooth a solid surface has to be for surface roughness not to have an effect on the contact angle.”

The combination W-DM calculated with the GM method provided fair *SFE* and δ results for rubber tree, blue gum leaves and pepper fruit. Most of the 2-liquid combinations and calculation methods generally led to γ^nd^_*s*_ under-estimations and sometimes γ^d^_*s*_ over-estimations for red iron bark, holm oak and Chilean myrtle compared to the 3-L results. The HM method generally led to the highest deviations from the 3-L method as compared to the GM method. According to Wu (1971, 1982) the HM approach has been described to be suitable for analysing surfaces with low *SFE* and not for applying it to systems involving phases with widely different polarizabilities. Limitations associated with the use of the HM method for assessing the SFE of surfaces having some degree of polarity have been reported earlier (e.g., Wu, 1971; Adão et al., 1999).

The results obtained for blue gum and holm oak leaves, and pepper fruit surfaces with the 3-L method and mean liquid surface tension values shown in Table 1, are within the range reported by Khayet and Fernández (2012) and Fernández et al. (2014a). The most remarkable difference is that we have considered the γ^+^_*l*_ of DM, which may be crucial for surfaces having significant polar components as noted by Tretinnikov (2000). The δ empirically determined for the smooth rubber tree and pepper fruit surfaces are coincident with the theoretical values estimated by Khayet and Fernández (2012), hence indicating a negligible effect of surface roughness and the prevalence of chemical interactions between such plant materials and liquid drops. On the contrary, the lower δ values determined for the rough iron bark, holm oak and to a lower degree, blue gum leaves reflect a significant effect of surface micro- and or-nano-structure on plant surface-liquid interactions.

## Conclusions

An analysis of the total surface tension and surface tension component values of test liquids reported in the literature led us to select the mean values for water (W), glycerol (G), and diiodomethane (DM), and enabled the comparison between different surface free energy calculation methods. Despite it would be less time-consuming to be able to measure only 2 liquids for characterizing plant surfaces of unknown physical and chemical properties, we gained evidence that it is not possible to a priori select a combination of 2 liquids and a suitable calculation method (GM or HM methods), which may provide results comparable to those obtained using the Lifshitz-van der Waals-acid-base method. We hence suggest the standard use of the gamma values calculated for W, G and DM (Table 1) and the 3-L method for drawing coherent conclusions concerning the surface free energy of plant surfaces, related for instance, to different species, organs, developmental stages or growing conditions.

### Conflict of interest statement

The authors declare that the research was conducted in the absence of any commercial or financial relationships that could be construed as a potential conflict of interest.
